# Decoding bioactive signals of the RNA secretome: the cell-free messenger RNA catalogue

**DOI:** 10.1017/erm.2024.12

**Published:** 2024-04-29

**Authors:** Rhys E. De Sota, Stephen R. Quake, John J. Sninsky, Shusuke Toden

**Affiliations:** 1 Superfluid Dx., 259 E Grand Avenue, South San Francisco, CA 94080, USA; 2 Department of Bioengineering and Department of Applied Physics, Stanford University, Stanford, CA, USA; 3 Chan Zuckerberg Biohub, San Francisco, CA, USA

**Keywords:** cell free messenger RNA, extracellular particles, extracellular vesicles, liquid biopsy, non-invasive biomarker

## Abstract

Despite gene-expression profiling being one of the most common methods to evaluate molecular dysregulation in tissues, the utilization of cell-free messenger RNA (cf-mRNA) as a blood-based non-invasive biomarker analyte has been limited compared to other RNA classes. Recent advancements in low-input RNA-sequencing and normalization techniques, however, have enabled characterization as well as accurate quantification of cf-mRNAs allowing direct pathological insights. The molecular profile of the cell-free transcriptome in multiple diseases has subsequently been characterized including, prenatal diseases, neurological disorders, liver diseases and cancers suggesting this biological compartment may serve as a disease agnostic platform. With mRNAs packaged in a myriad of extracellular vesicles and particles, these signals may be used to develop clinically actionable, non-invasive disease biomarkers. Here, we summarize the recent scientific developments of extracellular mRNA, biology of extracellular mRNA carriers, clinical utility of cf-mRNA as disease biomarkers, as well as proposed functions in cell and tissue pathophysiology.

## Introduction

The term ‘liquid biopsy’ refers to sampling and analysis of clinical fluids such as blood, saliva or urine for diagnostics as well as for monitoring disease and treatment (Ref. 1). Unlike the conventional tissue biopsy, liquid biopsy is non-invasive or minimally invasive and can be used to assess disease and/or health status of organs that are difficult to access. The term is somewhat of a misnomer since the information overlaps but is distinct from the RNA in cells and tissues in a ‘literal’ biopsy. Over the last two decades, circulating nucleic acids, cell-free DNA (cfDNA) in particular, have been utilized to develop clinically relevant biomarker assays for multiple diseases (Refs 2, 3). Measuring cfDNA permits the detection of genetic and methylation perturbations primarily from apoptotic or necrotic cells of various organs; however, many diseases do not result in genetic alterations (Ref. 4) and methylation (Ref. 5) only provides an indirect view of transcription. A potential strategy to directly survey dynamic transcriptomic bioanalytes is to survey extracellular RNA released from living cells.

**Figure 1.**
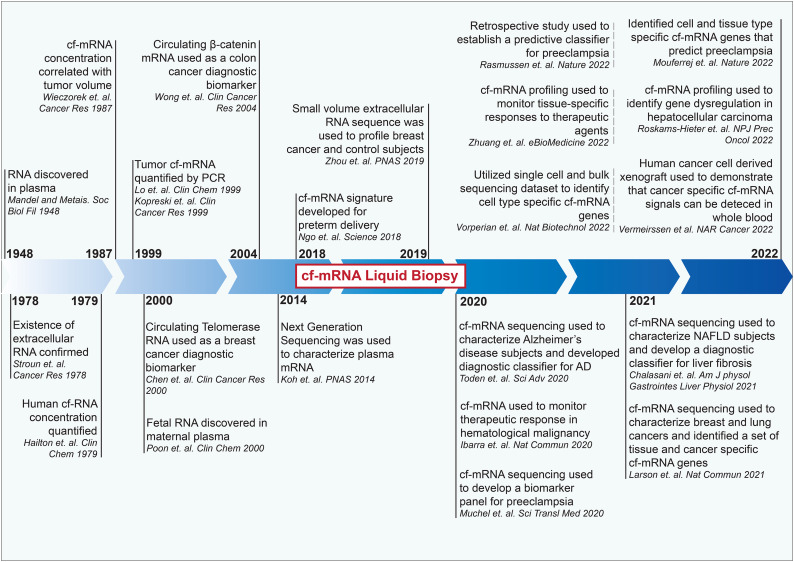
Chronological timeline of key studies in cf-mRNA liquid biopsy.

Although various types of cell-free RNA (cfRNA) are currently being investigated as possible biomarker candidates, the initial report of circulating cfRNA, RNA that exists outside of the blood cells in circulation, was first formally described in 1948 by Mandel and Metais (Fig. 1) (Ref. 6). Since the specific ribotypes were uncharacterized at the time of the cfRNA discovery, the attributes and functions of cfRNA were unknown. Measurement of targeted (hypothesis-dependent) cell-free messenger RNA (cf-mRNA) was first utilized as a disease diagnostic, biomarker analyte in 1999, when specific cancer associated gene transcripts were quantified in both plasma and serum (Refs 7, 8). In contrast, circulating microRNA (miRNA), a class of short non-coding RNA (ncRNA), was not identified until 2008 during a study examining placental RNA signal in plasma (Ref. 9). Despite this timeline, the application of cf-mRNA as a non-invasive disease biomarker analyte has lagged substantially compared to that of ncRNA counterparts (Ref. 10). Furthermore, the traditional approach of quantifying targeted genes by PCR was myopic, and only recently were unbiased (hypothesis-independent) strategies implemented, such as RNA-Sequencing, to survey all cf-mRNA combined with knowledge of cell and organ associated gene transcripts (Ref. 11). The limited utilization of cf-mRNA was also due to the low abundance of mRNA in the circulation and difficulties associated with quantifying these lowly expressed RNA transcripts. Cell free mRNA is encapsulated and/or bound in extracellular carriers rather than freely circulating, explaining why RNA, assumed to be highly labile to the ribonucleases (RNases) in biological fluids, could be detected (Ref. 12). NcRNAs such as miRNAs are able to form a stable complex with argonaut protein to evade RNase activity in the circulation (Ref. 13). One assessment of extracellular RNA content revealed the prominence of ncRNA, as miRNA and piwi interacting RNA (piRNA) occupy 40% of total RNA content, while mRNAs occupy only 2% of total extracellular RNA (Ref. 14). With recent application of unbiased nucleic acid quantification and cell source deconvolution techniques, however, an accurate quantification of rare circulating transcripts as well as comprehensive characterization of the cf-mRNA transcriptome has become feasible. With the recent recognition that extracellular nucleic acid plays a role in cell-to-cell communication and functions as indicator of disruption of cellular homeostasis, it is important to understand the biology of extracellular mRNA carriers. Extracellular mRNA carrier research describing the biogenesis, cargo, uptake, pathological contributions, and clinical applications has rapidly expanded over the past decade, providing numerous new archetypes and mechanisms of cf-mRNA carriers. For example, liquid–liquid phase separation has been implicated in the biogenesis of non-vesicle carriers (Ref. 15). Here we characterize extracellular cf-mRNA composition, various groups of extracellular mRNA carriers with proposed functions, and the contemporary clinical utilities as well as applications for cf-mRNA as a non-invasive health and disease biomarker.

## CF-mRNA characteristics and carriers

During the early stages of nucleic acid research, the presence of RNA degrading enzymes in blood led researchers to posit that cell-free RNAs would not be detected in the circulation. Thus, the first discovery of RNA in plasma and serum was enigmatic (Ref. 6). In 1978, Stroun *et al.* revealed that a portion of circulating RNA was protected from RNase activity via extracellular vesicle (EV) encapsulation (Ref. 16), demonstrating one of the key mechanisms by which cfRNA evades RNase digestion. The majority of exogenous, cell-free mRNAs were found to be digested in plasma/serum with an incubation time of just 15 s, bolstering the notion that free form mRNA is unlikely to exist in plasma/serum (Ref. 17). In contrast, endogenous RNA, those protected by EVs, were established as highly stable and minimally affected by freeze–thaw cycles even in delay of plasma processing for up to 24 h (Ref. 17). Subsequent studies showed that EVs are highly stable when stored at −80 °C (Ref. 18) and can withstand up to 3 months of prolonged heating at 37 °C (Ref. 19), further upholding EV-mediated mRNA stability.

Accumulating evidence indicates a disruption of cellular homeostasis that extracellular cell-to-cell communication plays a major role in disease aetiology (Ref. 20). Cells release a large amount of molecular materials that contribute to signals of homeostasis as well as direct or indirect intercellular signalling, and although EVs were initially considered to be cellular waste disposal mechanisms with little biological importance, they now are recognized as a key constituent in cell-to-cell communication mechanisms. This cell-to-cell communication also extends to extracellular particles (EPs), a more recently defined, broad class of extracellular RNA (exRNA) carriers. Defined together, extracellular vesicles and particles (EVPs) are excreted nanoparticles, released by almost every prokaryotic and eukaryotic cell, harbouring DNAs, mRNAs, ncRNAs, proteins, lipids, organelles and metabolites (Ref. 21). Most cell secreted vesicles and particles appear to enclose mRNAs, and importantly, EVPs can induce phenotypic perturbations on recipient cells. Delivery of genetic material via EVPs can be achieved through multiple mechanisms including receptor–ligand interactions, direct fusion of membranes and endocytic internalization (Ref. 22), and once EVs are internalized, horizontal genetic transfer takes place through various mechanisms such as cytoplasm targeting via endosomal fusion. In their review, Gruner and McManus emphasized the significance of intercellular communication involving extracellular RNAs (Ref. 23). Additionally, they underscored the necessity for standardizing extracellular RNA extraction protocols to improve comparability across studies.

One of the most interesting biological hypotheses involving EVP genetic transfer is the promotion of distant cancer metastasis through premetastatic niche formation (Ref. 24). Using a rodent model of metastasis, Hoshino and colleagues' landmark paper showed that a subgroup of cancer cell-derived EVs were taken up by specific organs, altering the molecular profile of host cells, and transforming tumour cells that normally lack the competency to metastasize in specific organs (Ref. 24). Furthermore, the functional role of EV-facilitated mRNA transfer was demonstrated in a rodent model of breast cancer (Ref. 25). In this study, transfer of vesicular RN7SL1 transcripts resulted in tumour growth, metastasis and therapeutic resistance, confirming that EVP-facilitated mRNA transfer plays an important biological role in oncogenesis. Transfer of miRNAs and lncRNAs through EVs have also shown to result in similar phenotypic alterations for cancer as well as other diseases (Refs 26, 27). Collectively, these studies set precedent for the biological importance of RNA cargos encapsulated in EVPs and have elevated EVP-facilitated genetic material transfer to the forefront of research in multiple diseases.

**Figure 2.**
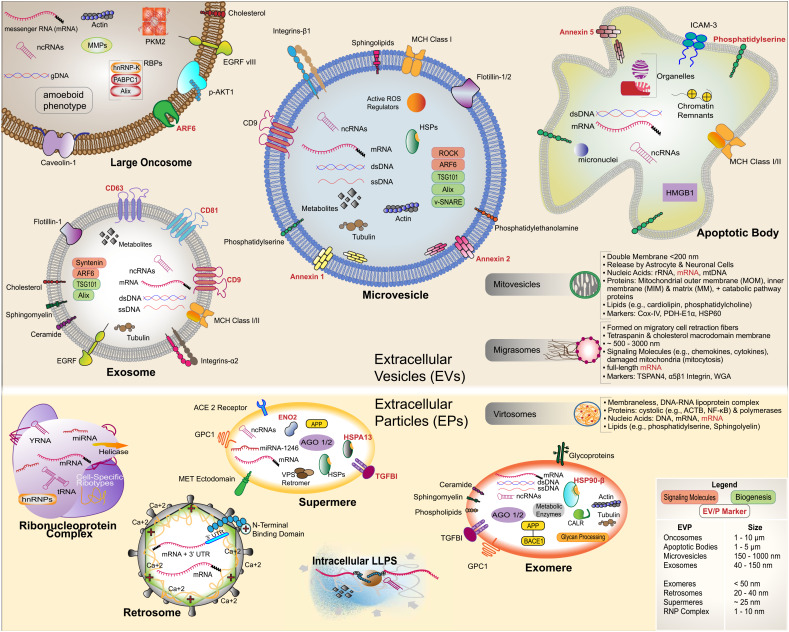
Key features of extracellular vesicles (EVs) and extracellular particles (EPs) (EVPs). EVs are shown in the top panel and EPs are shown in the bottom panel. Receptors are shown on the surface of EVPs. Key molecular contents are shown inside EVPs. The EVPs are not shown as scaled size. The sizes of EVPs are summarized in the figure legend.

Characterization of cf-mRNA carriers, however, has been confounded by a discordance of nomenclature (Ref. 28), an abundance of carrier subclasses among various biofluids and source cell types, a diversity in physical characteristics, and variable molecular cargo (Ref. 29). While a common division of cell released extracellular materials is extracellular vesicles (EVs) and extracellular particles (EPs), that oversimplifies the diversity of extracellular RNA carriers. Rather than delineating each extracellular archetype, this review will consider ‘extracellular vesicles’ as a generalized term to represent, lipid-bilayer-delimited particles released from a cell, while ‘extracellular particles’ delineate non-lipid-bilayer extracellular RNA carriers (Ref. 29). Broad classification of mRNA containing EVPs are large EVs (>200 nm) including apoptotic bodies, large oncosomes and microvesicles, small EVs (<200 nm) encompassing exosomes and ARRDC1-mediated microvesicles (ARMMs) as well as EPs such as exomeres, supermeres, retrosomes and ribonucleoprotein complexes (Fig. 2). The key challenge of extracellular RNA carrier classification is the innate heterogeneity that exists within and between these two overall categories. For example, Zhang *et al.* postulated that exosomes can be stratified into 60–80 nm small exosomes (Exo-S) and 90–120 nm large exosomes (Exo-L) due to their apparently distinct proteomic profiles (Ref. 30). Other than size, characterization has also been parsed into density, content/cargo, biogenesis, functional impact on the recipient cell, and cell of origin, all of which impact the biomarker and tropistic capabilities of each EVP (Ref. 31). We identified key EVPs that are known to contain mRNAs or those EVPs that most likely contain mRNAs, and summarized the proposed biogenesis, cargo and potential mechanisms of RNA loading for extracellular release (Table 1). As extracellular mRNA may serve as a significant clinical biomarker source in neurodegeneration, oncology and other fields, interrogating presence, delivery and biological impact may lead to the development of clinically relevant biomarkers and identification of potential therapeutic targets (Refs 21, 32).

**Table 1.** Key characteristics of extracellular vesicles and particles

Carrier name	Type	Encapsulation	Size (nm)	LLPS	Release mechanism	Key features	Surface protein marker (s)	Isolation method(s)	mRNA identified	Refs
Exosomes	EV	Lipid bilayer	40–50	No	Plasma membrane shedding	Oncogenic protein transfer, tumour potentiation and promotion, fibroblast education, viral infection, immune modulation, pathological environment progression	CD63, CD81, CD9	Density gradient ultracentrifugation, size-exclusion chromatography, immunoaffinity capture methods, HPLC, high-speed exosomal pelleting, fluorescence-activated sorting, EXODUS	Yes	(Refs 31, 33, 34, 35, 36, 37, 38)
Microvesicles	EV	Lipid bilayer	150–1000	No	Plasma membrane blebbing	Prion-RNA dissemination, oncogenic protein transfer, tumour potentiation and promotion, immune modulation, pathological environment progression, cell-specific mRNA transfer	Annexin 1 & 2	Density gradient ultracentrifugation, high-resolution density gradient fractionation, size-exclusion chromatography, immunoaffinity capture methods, HPLC, polymer precipitation	Yes	(Refs 36, 37, 39, 40, 41)
ARMMs	EV	Lipid bilayer	≈50	No	Plasma membrane blebbing	Therapeutic macromolecule delivery (e.g., p53) correlated to ARRDC1 expression	Ub-ARRDC1, TSG101	Immunoaffinity capture, serial ultracentrifugation, sucrose gradient fractionation	Yes	(Refs 42, 43)
Large Oncosome	EV	Lipid bilayer	1000– 10 000	No	Plasma membrane blebbing	Oncogenic protein transfer, amoeboid cell phenotype, metastatic potentiation	Annexin A1, CK18, ARF6, V-ATPase G1	Ultracentrifugation	Yes	(Refs 44, 45, 46)
Apoptotic Body	EV	Lipid bilayer	1000–5000	No	Plasma membrane blebbing	Apoptotic clearance, antigen immune activation, phagocyte promotion	Annexin V	Differential centrifugation, sucrose gradient centrifugation	Yes	(Refs 47, 48, 49)
Migrasomes	EV	Lipid bilayer	500–3000	No	Retraction fibre tetraspanin microdomains	Damaged organelle removal, combinatory immune signalling, maintenance of cellular homeostasis	TSPAN4, α5*β*1, Integrin, WGA	Density-gradient centrifugation, culture dish adherence	Yes	(Refs 32, 50, 51)
Mitovesicles	EV	Lipid bilayer	<200	No	Mitophagy triggered plasma membrane shedding	Mitochondrial DNA/RNA/Proteins, neurotransmitter modulation, removal of dysfunctional mitochondria and ROS species	Cox-IV, PDH-E1α, HSP60	High-resolution density gradient	Yes	(Ref. 52)
Retrosomes	EP	Single-lipid membrane	20–40	No	Secretory exocytosis	Endogenous viral-like capsid, highly expressed in neurons, co-opted gag genes, self-assembling	Viral envelope, Gag, viral-epitope specific, Arc capsid	Size-exclusion chromatography, iodixanol gradient ultracentrifugation, immunoprecipitation, gradient ultracentrifugation	Yes	(Refs 53, 54, 55)
Exomeres	EP	Amembrous	<50	Yes	Secretory exocytosis	Metabolic process proteins, oncogenic signalling, tumour proliferation	ApoM, HSP90, LGALS3BP, FASN, ACLY	Asymmetric flow field-flow fractionation, Iodixanol density gradient fractionation, sequential ultracentrifugation	No	(Refs 30, 36, 56, 57)
Supermeres	EP	Amembrous	≈25	Yes	Secretory exocytosis	Clinically relevant proteins, cross blood–brain barrier, concentrated exRNA + RNPs	TGFBI, HSPA13, ENO2	Sequential ultracentrifugation, density gradient fractionation	No	(Refs 37, 58, 59)
Ribonucleoprotein particles	EP	Amembrous	3–10	No	Secretory exocytosis, plasma membrane shedding, intra-EVP secretion	EVP RNA loading, viral RNA dissemination, immune regulation	Protein-specific complex, RNA-binding domains	Antibody capture, GE, polypeptide capture, chromatography, sequential filtration	Yes	(Refs 60, 61, 62)
Virtosomes	EP	Amembrous	<20	Yes	Secretory exocytosis	DNA, RNA, and lipoprotein complex. Modifies recipient cells.	Cell-specific	Cs_2_SO_4_/CsCl gradient centrifugation, Sucrose gradient centrifugation	No	(Refs 63, 64)
Nucleocapsid	EP	Amembrous	3–30	Yes	Secretory exocytosis	Amembrous endogenous viral-like capsid, highly expressed in neurons, co-opted gag genes, self-assembling	Viral envelope, Gag, viral-epitope specific	Size-exclusion chromatography, iodixanol gradient ultracentrifugation	Yes	(Ref. 65)

EV, Extracellular vesicles; EP, extracellular particles; EVP, extracellular vesicles and particles.

### Extracellular vesicles

#### Exosomes

Exosomes are 40–150 nm, lipid-bilayer membrane-bound particles that result from plasma membrane shedding and are currently the most well-studied EV type. The biogenesis consists of a double invagination of the plasma membrane with multivesicular bodies (MVBs) that contain intraluminal vesicles (ILVs). ILVs selectively incorporate DNA, RNA, metabolites, proteins and lipids and are thought to be precursors to endosomes. MVBs fuse with the plasma membrane and the ILVs are released into extracellular space as exosomes (Refs 31, 39). Putatively, exosomes have been shown to be involved in pathways associated with Ras-related protein GTPase Rabs, Syntenin 1, TSG101, ALIX, syndecan-1, tetraspanins, glycophosphatidylinositol-anchored proteins, SNAREs, flotillin and MHC I/II molecules (Refs 31, 40). The canonical ESCRT pathway drives microdomain formation in MVBs and ILVs, however, exosomes can also be formed in an ESCRT independent pathway (Ref. 39).

The classical exosomal surface protein markers are CD63, CD81 and CD9 (Refs 39, 40) and exosomes likely undergo a tightly regulated loading process due to the lack of cytoskeleton elements such as microfilaments, microtubules, intermediate filaments and highly abundant cytosolic enzymes (Ref. 40). For more on exosomes, several excellent reviews comprehensively summarized exosome biogenesis, signals and markers (Refs 31, 33, 34, 39, 40). Notwithstanding, the categorization of exosomes merit further grouping, since, for example, there are several types of EVs that are known to utilize the MVB biogenesis pathway but lack traditional tetraspanin markers (Ref. 40).

The revelation of exosome-mediated mRNA transfer as a mechanism for cell-to-cell communication and distal cell modulation was first theorized in 2007 (Ref. 66). Valadi *et al.* investigated the functional roles of extracellular carriers derived from murine and human mast cell lines and subsequently demonstrated recipient cell uptake of functional mRNAs that were capable of translation (Ref. 66). Additionally, a study using a transgenic mouse model demonstrated exosomal mRNAs being transferred from haematopoietic cells to Purkinje neurons (Ref. 67). Exosomal mRNAs have also been found to be released by and incorporated into various human cancer cell lines (Ref. 68). In a murine model, exosomes that were derived from a metastatic mammary tumour increased recipient cell metastatic and migratory potential upon mRNA transfer (Ref. 35). It has also been proposed that since exosomes carry the 3′-UTR of mRNAs, recipient cell uptake of these regions can induce gene expression alteration, protein translation and subcellular localization of mRNAs (Ref. 69).

#### Microvesicles

Microvesicles are another classical EV that are released into the extracellular space through direct outward budding of the plasma membrane followed by a tightly regulated pinching and scission process (Ref. 41). These EVs range in size from 150–1000 nm and the biogenesis involves plasma membranous lipid and protein rearrangement, especially cholesterol and ceramide recruitment, along with altered Ca^2+^ levels (Ref. 39). Proteins responsible for membrane reshaping include TSG101, Vps4 and the Ca^2+^ dependent enzymes flippases and floppases, while ARF6-induced actomyosin contractility and other small GTPases RhoA, Cdc42, and Rac1 regulate abscission and shedding (Refs 39, 40, 70). Targeting of TSG101 to the plasma-membrane occurs via an interaction with ARRDC1, a localization that does not occur in MVBs, potentially providing a different mechanism for TSG101 between exosomes and microvesicles (Ref. 36). Furthermore, through high-resolution density gradient fractionation and direct immunoaffinity capture, Annexin 1 has been identified as a specific protein marker for canonical microvesicles (Ref. 40). While the specific mechanisms for nucleic acid targeting to the plasma membrane and for packaging into microvesicles are still unelucidated, a conserved zipcode-like 25-nt sequence in the 3′UTRs of many microvesicle-enriched mRNAs has been identified in human cancer cells. This zipcode RNA sequence motif contains a miR-1289 binding site that orchestrates mRNA transfer into microvesicles (Ref. 71). Along with this RNA sequence motif that provides a framework for mRNA packaging, other sequence motifs, secondary configurations, wide-ranging affinities for various lipids and RNA binding proteins, RNA modifications, and ncRNA composition likely play roles in mRNA localization and packaging (Ref. 21).

Early reports indicated that tumour-derived microvesicles contained mRNA and were able to modulate oncogenic signalling pathways through horizontal transfer (Ref. 72). Subsequently, the landmark study of Skog *et al*., demonstrated that microvesicles from glioblastoma cells harboured mRNAs functionally associated with angiogenesis, cell proliferation and maintenance of the human glioma cell line (Ref. 73). Although these early studies did not perform appropriate purification procedures to differentiate exosomes, microvesicles and non-vesicular fractions, canonical microvesicles from the studies appear to have manipulated tumour microenvironments by inducing phenotypic changes as well as enhancing tumour growth and invasion (Ref. 73). Furthermore, another study reinforced that microvesicles from therapy-resistant glioblastoma stem-like cells contained both fragmented and full-length mRNA transcripts. These results were then replicated in glioblastoma-derived microvesicles and common glioblastoma mutated transcripts, such as PTEN and COL1A2, were detected. Studies have noted that extracellular RNA is not reflective of intracellular RNA (Ref. 66), but it is noteworthy that in this glioblastoma study, the authors found microvesicles to be a closer reflection of cellular RNA composition than exosomes and extracellular ribonucleoproteins (Ref. 60). This has applicable implications for microvesicles as an analyte in clinical practice since the molecular profile of microvesicles could be used to survey cell states and somatic mutations.

#### ARMMs

In addition to canonical microvesicles ranging from 150 to 1000 nm in size, there are also reports of *ARRDC1-mediated microvesicles (ARMMs)* that average 50 nm in size. Mirroring microvesicle biogenesis, ARMMs biogenesis consists of TSG101 localization to the plasma membrane by ARRDC1, E3 ligases ubiquitinating ARRDC1 and VPS4 ATPase catalysing ARMMs' scission from the plasma membrane (Ref. 42). Aside from size, it is unknown if ARMMs are distinct from canonical microvesicles, possibly being a variant induced by uncharacterized mechanisms or produced by certain cell types. In clinical applications, because of the known reliance on ARRDC1 for ARMM's budding as well as the elevated release of ARMMs in proportion to ARRDC1 overexpression, ARMMs have been used as a vehicle to deliver exogenous, tumour suppressing P53 mRNAs to recipient cells in a *in vivo* experiment (Ref. 43). This study highlights the potential utility of ARMMs for specific delivery of bioactive molecules and precision therapies.

#### Large oncosomes

Large oncosomes are another class of membrane blebbing EVs. These non-apoptotic blebs originate through membrane shedding from cancer cells with an amoeboid phenotype, and they are atypically large ranging from 1 to 10 *μ*m in diameter. Increased large oncosome shedding and promoted phenotypic alterations occur through activation of key oncogenic pathways. For example, large oncosomes derived from a pancreatic cancer cell line were found to reprogram normal pancreatic fibroblasts, stimulate endothelial tube formation and promote tumour growth *in vivo* (Ref. 44). Similarly, an *in vitro* study indicated that pancreatic cancer cell line derived large oncosomes activate the Akt pathway and subsequently enhance proliferation and migration potential of recipient tumour cells (Ref. 45). Large oncosomes are highly bioactive and have been shown to harbour mRNA processing and translation factors, such as ARF6, that increase mRNA expression and promote tumour invasion (Ref. 74). Because they originate from aggressive cancer cells with an amoeboid phenotype, large oncosomes may play a key role in progressive remodelling of tumour microenvironments as well as metastatic progressions, termed ‘oncosome-mediated remodeling’ (Ref. 46). Further research is needed to differentiate the molecular characteristics of large oncosomes from tumour derived, canonical MVs, but the atypical size of oncosomes has the potential to correspond with a higher abundance of functionally relevant EV cargo.

#### Apoptotic bodies

Apoptotic bodies are heterogeneous, 1–5 *μ*m extracellular vesicles that originate from cell fragmentation during apoptosis, a process that occurs in over 200 billion cells in the human body every day (Ref. 75). There are a wide variety of stimuli to induce apoptosis, both physiological and pathological, and the proposed functions of apoptotic bodies are clearance of apoptotic debris, intercellular communication, and immune regulation (Ref. 47). Once apoptosis is induced, a caspase-dependent proteolytic cascade is activated (Ref. 48), particularly with ROCK1 and PANX1 acting to regulate membrane blebbing and apoptopodia formation (Ref. 75). Additionally, apoptotic cells and apoptotic bodies exhibit chemoattractive, ‘find me’, signals such as ATP, UTP and CX3CLI/fractalkine, as well as phagocyte recognizing, ‘eat me’, signals with partiality towards exposure of phosphatidylserine on the outer leaflet of the plasma membrane as well as ICAM-3 (Ref. 47). Annexin V binds to phosphatidylserine on the outer leaflet of the plasma membrane and is the most common marker for apoptotic bodies (Ref. 33). Although the exact mRNA content of apoptotic body remains unclear, RNAs that are contained in both apoptotic cells and apoptotic bodies have been reported to possess short half-lives (Ref. 76). In addition, cell death has been shown to trigger global mRNA decay (Ref. 77), but further research should be conducted to determine how much, if any, mRNA escapes into the extracellular environment.

### Other extracellular vesicles

There is a slew of other EVs that contain functional mRNA and can promote cell-to-cell communication. *Migrasomes* are approximately 2 *μ*m EVs that form on retraction fibres of mobile cells through tetraspanin-enriched macrodomains, specifically marked by tetraspanin-4 duplexed with cholesterol (Ref. 50). These EVs contain signalling molecules that, when released, mediate ligand–ligand cell communication proximally or distally, help dispose damaged mitochondria in cells experiencing mild mitochondrial stress, termed mitocytosis, and are enriched in translationally competent, full-length mRNAs (Ref. 51). While horizontal transfer of mRNA from migrasomes to recipient cells has been demonstrated *in vitro*, an additional *in vivo* validation is needed to elucidate this mechanism. *Mitovesicles* are double-membraned, electron-dense EVs, typically smaller than 200 nm in diameter. These EVs are uniquely enriched in mitochondrial proteins, mtDNAs, mtRNAs, and mitochondrial encoded mRNAs released by cerebral neurons and astrocytes (Ref. 52). Importantly, the authors showed upregulated mitovesicle release and downregulated mitovesicle mRNA levels in post-mortem Down Syndrome brains and murine Down Syndrome models compared to diploid control brains, highlighting potential involvement in disease. More recently, *midbody remnants* of cell division have been reported to not only fuse with one of the daughter cells, but also be released as extracellular vesicles (Ref. 78). These studies reported protein profiling of midbody remnants from cell lines and their influence when exposed to fibroblasts. Further studies will be necessary, however, to determine whether the resulting alterations in fibroblast phenotype requires internalization alone, or if this novel EV acts in a combinatory process.

### Extracellular particles

#### Exomeres

Exomeres are recently discovered non-membranous, nanoparticles that arise from secretory exocytosis and contain selectively incorporated DNA, RNA, lipids and metabolites. Zhang *et al*. used asymmetric flow field-flow fractionation to isolate exomeres and were subsequently the first to describe this particle (Ref. 30). Using atomic force microscopy, exomeres from peripheral blood were measured to be approximately 3 nm in length and 30 nm in diameter (Ref. 79). Currently, the specific markers for exomeres are Apolipoprotein M, HSP90 and LGALS3BP (Refs 28, 30). Exomeres lack proteins associated with exosome and microvesicle biogenesis, and instead are enriched in proteins associated with the endoplasmic reticulum, mitochondria and microtubules, all of which could be involved in the biogenesis of exomeres (Ref. 36).

In the subsequent study, Zhang *et al.* separated exomeres from exosomes using sequential ultracentrifugation (Ref. 56). Proteomic profiling of exomeres showed that these particles are highly enriched in glycolysis enzymes, Argonaute 1/2 and proteins involved in Alzheimer's Disease pathology including APP, APPL2 and CLSTN 1–3 (Ref. 56). Additionally, exomeres appear to be functionally active as they encapsulate ST6Gal-1 and AREG which can hypersialylate membrane proteins such as *β*1-integrin and modulate EGFR trafficking in recipient cells to enhance tumour organoid function, respectively (Ref. 56). Furthermore, shed ectodomain fragments of ACE2 and DPP4 are enriched in exomeres, two key receptors for SARS-CoV1, SARS-CoV-2 and MERS-CoV. These α2,6-sialylated ACE2 exomeres were able to bind to SARS-CoV-2 S1-protein subunits and were hypothesized as decoys to attenuate infection (Ref. 57). For nucleic acids, the DNA in exomeres was found to be evenly distributed in a broad range of sizes between 100 bp–10 kb and exomeres have the highest relative levels of miRNAs and other small RNAs (Refs 30, 58). Of note, exceptionally high levels of full-length rRNAs were found in exomeres compared to other EPs (Ref. 28). Considering that rRNA interferes with quantification of mRNA, strategies such as ribodepletion and exome enrichment will most likely enhance possible mRNA signals. Further research will be important to explicate mRNAs prevalence in exomeres, elucidate cargo and packaging mechanisms, uncover biogenesis pathways and answer questions regarding the functions of exomeres, both physiologically and pathologically.

#### Supermeres

Supermeres are another recently identified nanoparticle discovered in the ‘supernatant of exomeres’ following pelleting of exomeres via ultracentrifugation (Ref. 58). Like exomeres, supermeres are membraneless nanoparticles but are of smaller size, approximately 25 nm. Due to similar molecular profiles between exomeres and supermeres, it was first speculated that these two nanoparticle types are continuous populations of nanoparticles (Ref. 28), however, reanalysis of the original Zhang *et al*. data indicated a substantial number of differentially expressed proteins between the groups (Ref. 80). Many disease-associated proteins are enriched in supermeres including APP, MET, GPC1, AGO2, ACE, ACE2 and TGFBI, highlighting the potential involvement of supermeres in cancer, neurological disorders, heart disease and COVID-19. Further, in an *in vitro* study of a human colorectal cancer cell line, authors observed drug resistance and increased lactate secretion, a hallmark of the Warburg effect, after supermere uptake by recipient cells (Ref. 58). Subsequently, treatment of cells with bafilomycin A, an inhibitor of autophagy, caused inhibition of supermere accumulation, pointing to micropinocytosis as the uptake mechanism for supermeres (Ref. 58). Of note, the heat-shock protein HSPA13 was enriched in supermeres from five different cell lines, suggesting it may be an actionable marker protein in future studies (Ref. 58). One of the key characteristics of supermeres is the substantially high RNA content compared to exomeres and small EVs, and supermeres are enriched in RNA binding proteins such as YBX1, sumoylated hnRNPA2B1, and argonaute proteins (Ref. 58). Given that these RNA binding proteins have previously been shown to mediate exosomal mRNA secretion, as well as the high concentrations of RNA, there may be corollaries between the presence of mRNA in exosomes and supermeres. More research is warranted for this novel EP, and with supermeres appearing to cross blood–brain barrier more readily than exomeres and small EVs (Ref. 58), they are a promising analyte in the development of neurological disease biomarkers as well as a vehicle to deliver therapeutic molecules across the blood–brain barrier.

#### Retrosomes and nucleocapsids

Retrosomes are a unique, 20–40 nm diameter EP that are characterized by self-assembly into protein capsids of ancient conscripted nucleocapsid genes. These retrosomes retain parallels to their Gag precursor proteins encoded by ancient retroviruses and retrotransposons including self-assembly into oligomeric capsids, RNA encapsulation, RNase resistance and intercellular transmission of RNA (Ref. 53). A well-studied example is the neuronal gene *Arc*, which has been shown to act as a retrosome in neurons by self-assembling into a mRNA containing virus-like capsids (Ref. 53). Structurally, *Arc* mRNA induced capsid formation and transfer is likely regulated through a 200 amino-acid N-Terminal domain capable of binding RNA and negatively charged membranes (Ref. 81). Retrosomal *Arc* mRNA is transferred from neuron to neuron in the central nervous system (Ref. 53), and this functionally regulates memory consolidation through synaptic plasticity, transcriptional regulation and neuronal bouton to bouton transmission. Similarly, the drosophila *dArc1* protein associates with the 3′ UTR of its own mRNA across synapses to promote synapse maturation and plasticity (Ref. 54). Uptake and transfer of *Arc* retrosomes occurs through endocytosis, comparable to other EVPs, but retrosomes are unique because of a single lipid membrane, and more closely resembles non-enveloped viral RNA transfer mechanisms (Ref. 53). Nevertheless, it is still unknown whether release of retrosomes occurs in a manner resembling viral hijacking of ESCRT machinery in HIV (Ref. 82) or alike non-enveloped viruses such as leishmania RNA virus 1, hepatitis A virus and hepatitis C virus (Ref. 21).

Similarly, nucleocapsids are the other recently discovered viral-like EP that is Gag-like and evolutionarily repurposed from retrotransposons. Although nucleocapsids are noticeably stable under high concentrations of detergent, they degrade in the presence of proteinase K highlighting a lack of membrane protection and the key differentiation from lipid-encased retrosomes. A recent study highlighted that the PNMA2 protein, a highly conserved retrotransposon element in placental carrying mammals, is responsible for the release of 20–30 nm nucleocapsids. PNMA2 is principally concentrated in human brain tissue samples and PNMA2 mRNA is predominantly expressed in excitatory and inhibitory neurons. Importantly, the authors hypothesized that PNMA2 nucleocapsid expression outside of the central nervous system may be a key effectuate in paraneoplastic neurological syndromes, especially because paraneoplastic autoantibodies were found to be highly specific to nucleocapsid surface spike epitopes (Ref. 65). There is an abundance of retroelements in human genome, and given that this is an evolutionary conserved mechanism, there are likely other functionally relevant retrosomes and nucleocapsids waiting to be uncovered.

### Other extracellular particles

*Ribonucleoproteins* (RNPs) are well-known, key contributors to EVP organization; however, they also exist as extracellular, non-vesicular RNP complexes. These complexes are concentrated with small regulatory RNAs including miRNA (Ref. 83), tRNA and yRNAs (Ref. 84), and have an RNA signature readily distinguishable from EVs. The mRNA fraction of extracellular RNPs derived from glioblastoma cells was shown to be less than 5% of total RNP RNA (Ref. 60), but an improvement in RNP purification methods are needed to further solidify the composition of RNPs (Ref. 84). Finally, *virtosomes* (Ref. 63) and *SMAPs* (supramolecular attack particles) (Ref. 85) are both EPs that potentially, and a priori contain mRNA but warrant further investigation.

## EVP formation and organization

### Liquid–liquid phase separation and biomolecular condensates

Amembrous EPs such as supermeres, exomeres and ribonucleoproteins, as well as contents within EVPs are part of a highly orchestrated, intracellular sorting and signalling network that is induced, in part, through liquid–liquid phase separation (LLPS) (Refs 86, 87). Putatively, LLPS can be defined as the formation of two immiscible fluids from a single homogeneous mixture (Ref. 88), and LLPS forms membraneless assemblies of concentrated biomolecules called ‘biomolecular condensates’ (Ref. 89). Condensates are crucially important in cellular organization as they help biophysically manage the localization of reaction components, cellular concentration equilibria, and compartmentalization of specific molecules (Ref. 89). Additionally, biomolecular condensates potentially provide a framework for the organization of freely diffusive, yet specific pathways associated in EVP biogenesis and packaging. For instance, in larger cells it is crucial to enclose mRNAs in condensates for long-range transport and to ensure localized and specific translation or packaging (Ref. 88). This control of biochemical flux is often dependent on RNA through binding to multivalent, disordered residues of RNA binding proteins, which are regions of RNA-binding proteins that allow multiple elements to govern molecular interactions, subsequently leading to demixing of phases through opposite charges. Other properties that govern phase separation dynamics and potential include RNA modifications, alternative splicing, RNA–RNA interactions, and individual cell transcription profiles (Ref. 88). In one study, Zhang *et al.* demonstrated that specific mRNA targets of the RNA-binding protein Whi3 altered the viscosity of droplets, fusion kinetics and bulk solution exchange rates, importantly showing that mRNA not only encodes genetic information but imparts distinct biophysical properties on condensates (Ref. 90). Along with potentially deciphering EVP biogenesis and organization, LLPS is also associated with many pathologies. RNA-binding proteins that can phase separate alter biomolecular condensate characteristics based on specific binding molecules, otherwise called polyphasic linkage. This shift of phase behaviour through the binding of specific nucleic acids and/or proteins may lead to pathological conditions (Ref. 91). In AD and other neurological disorders, for example, aberrant phase transition through the dysregulation of RNA binding leads disordered protein regions to form amyloid-like fibres (Ref. 92) and phosphorylated or mutant Tau induces phase separation that promotes aggregation (Ref. 91). Further research should focus on the role of LLPS in EVP biogenesis, packaging and roles in disease. LLPS concentrates interaction partners in biological pathways, while excluding other components. This framework may be foundational to understand the underlying mechanisms of many convoluted EVP biogenesis networks, especially when differential mechanisms and kinetics occur in pathological conditions.

### RNA binding proteins

Concomitantly to LLPS, RNA-binding proteins are emerging as critical factors in the interactome of EVP biogenesis and cargo loading, and it is widely accepted that RNA-binding proteins are implicated in the active sorting of various ribotypes (Ref. 61). For example, hnRNPA2B1 controls the transport and subcellular location of mRNAs in neurons (Ref. 93) and is responsible for sorting specific miRNAs into exosomes and microvesicles (Ref. 61). Additionally, sumoylations of hnRNPA2B1 are preferential in exosomes (Ref. 94) and sumoylated hnRNPA2B1 is enriched in supermeres, all of which points to hnRNPA2B1's likely role in certain EVP biogenesis mechanisms. YBX1 is complexed with TSG101 in the interactome of the ESCRT pathway, while silencing of the MVP gene in HEK293F cells has been shown to substantially decrease mRNA loading into exosomes (Ref. 95). Xu *et al.* uncovered that hnRNPH1-mRNA levels in exosomes purified from serum are remarkably higher in hepatocellular carcinoma patients compared to healthy subjects, indicating that hnRNPH1 may be a potential biomarker candidate in hepatocellular carcinoma diagnosis (Ref. 96). RNA-binding proteins are well known factors in nucleic acid metabolism, alternative splicing and translation regulation, and ostensible mediators of EVP biogenesis, trafficking and cargo loading. Considering that RNA governs the properties of LLPS and RNA-binding proteins (Ref. 97), it will be important in the future to understand mechanisms such as RNA-binding protein -mRNA binding patterns and selective RNA-binding protein enrichment in EVP subtypes. It is postulated that LLPS and RNA-binding proteins are not distinct, but rather synergistic in modulating properties and functions of EVPs, and the divergence of these processes likely leads to pathological states such as neurodegeneration (Ref. 97).

## Utilization of cf-mRNA as non-invasive disease biomarkers

**Figure 3.**
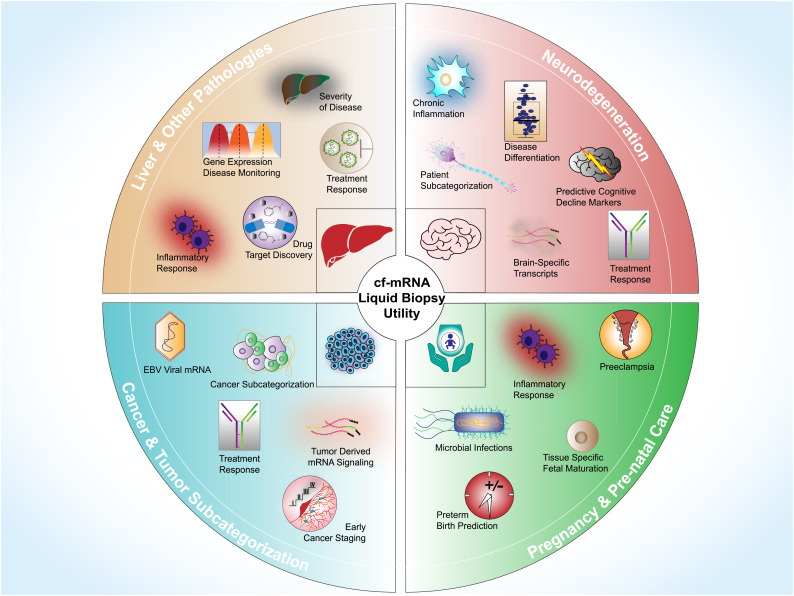
Clinical utility of cf-mRNA in liquid biopsy. Examples of how cf-mRNA could be used in neurodegenerative disease (top right), pregnancy and pre-natal care (bottom right), cancer and tumour subcategorization (bottom left) and liver/other pathologies (top left).

Although there are several clinically relevant cfDNA-based disease biomarkers, development of cfRNA-based clinical biomarkers has been sluggish. Apart from the fundamental differences between DNA and RNA, accumulating evidence indicates that the origin of cfDNA and cf-mRNA may also be distinctively different. Contra to cf-mRNAs that predominantly exist within tropistic extracellular carriers (Ref. 12), a recent study indicated that only a fraction of exosomes contain genomic DNA (Ref. 98) and cfDNA is postulated to be mainly sourced from apoptotic cells and bodies (Refs 99, 100). These differences in origin will be key factors that need to be considered when developing disease-specific blood-based biomarkers. There are several ncRNA-based liquid biopsy candidates currently being investigated, with miRNA, in particular, gathering much attention (Ref. 10). However, in comparison to the estimated 24 000 total human coding genes (Ref. 101), the human genome only encodes for approximately 2600 mature miRNAs (Ref. 102). Additionally, establishing the functional role of individual miRNAs is complex as they bind to 3′-untranslated regions of several hundred target genes, regulate multiple cellular processes by modulating RNA translation, and multiple miRNAs can bind to the same mRNA. In contrast, the functional roles of many coding genes are well-established, and there are also several highly characterized sequencing references for tissue- and cell-type mRNA expression (Refs 103, 104, 105, 106, 107, 108). Comparing gene-expression profiles between organs has been useful to reveal various subsets of mRNA transcripts highly specific to organs that can be used as unique identifiers to establish tissues of origin (Ref. 104). Similarly, a large set of human cell types have been characterized using single cell RNA-sequencing (Ref. 105). By examining cell types of origin with the Tabula Sapiens transcriptomic cell atlas along with tissue sequencing datasets, a recent study demonstrated that it is possible to decompose the cf-mRNA transcriptome at the cell-type level (Ref. 109). Other RNA species including lncRNA and piRNAs have greater diversity than mRNAs, 60 000 (Ref. 110) and 30 000 (Ref. 111) genes, respectively, but these genes have not been annotated and their functional roles remain uncertain. Thus, the large selection of well-characterized mRNA genes and comprehensive datasets that enable tissue- and cell-type origin gives mRNA an advantage over other cfRNA types as a blood-based biomarker analyte. Here, we summarized several key biomarker studies that utilized cf-mRNA as an analyte (Table 2) and discussed how cf-mRNA profiling could be incorporated into biomarker development strategies (Fig. 3).

**Table 2.** Summary of key cf-mRNA clinical studies

cf-mRNA biomarkers	Disease/condition type	Content	Platform	Key outcomes	Published year	Refs
**Prenatal biomarkers**						
Poon *et al*.	Pregnancy	Plasma	PCR	Identified the presence of fetal RNA in maternal plasma.	2000	(Ref. 112)
Koh *et al*.	Pregnancy	Plasma	microarray / RNA-Seq	Monitored gene expression alternations during various trimesters of pregnancy.	2014	(Ref. 11)
Pan *et al*.	Infection during pregnancy	Plasma	RNA-Seq	Demonstrated that cf-mRNA profiling could be used to monitor immune response and microbial infection during pregnancy.	2017	(Ref. 113)
Ngo *et al*.	Preterm delivery	Plasma	RNA-Seq	Demonstrated the feasibility of cf-mRNA-based biomarker for preterm delivery.	2018	(Ref. 114)
Munchel *et al*.	Preeclampsia	Plasma	RNA-Seq	Identified cf-mRNA biomarkers for early-onset preeclampsia.	2020	(Ref. 115)
Moufarrej *et al*.	Preeclampsia	Plasma	RNA-Seq	Identified 18 cf-mRNA gene biomarkers for high-risk preeclampsia.	2022	(Ref. 116)
Rasmussen *et al*.	Preeclampsia	Plasma	RNA-Seq	Identified a cf-mRNA gene-signature that can predict preeclampsia.	2022	(Ref. 117)
**Cancer biomarkers**						
Chen *et al*.	Breast cancer	Serum	PCR	RNA subunits of telomerase were detected in breast cancer serum.	2000	(Ref. 118)
Wong *et al*.	Colorectal cancer	Plasma	PCR	Identified plasma beta catenin as a potential colorectal cancer diagnostic biomarker.	2004	(Ref. 119)
Zhou *et al*.	Breast cancer	Serum	RNA-Seq	Identified several differentially expressed cf-mRNA genes between cancers and healthy controls.	2019	(Ref. 120)
Ibarra *et al*.	Haematological cancers	Plasma	RNA-Seq	Demonstrated the feasibility of cf-mRNA profiling as an approach to monitor treatment responses of haematological malignancies.	2020	(Ref. 121)
Souza *et al*.	Prostate cancer	Plasma	PCR	Identified a panel of cf-mRNA genes that can distinguish prostate cancers from healthy controls.	2020	(Ref. 122)
Larson *et al*.	Breast and lung cancers	Plasma	RNA-Seq	Identified tissue type-specific cf-mRNA biomarkers in breast and lung cancers.	2021	(Ref. 123)
Roskams-Hieter *et al*.	Hepatocellular carcinoma and multiple myeloma	Plasma	RNA-Seq	A small panel of cf-mRNA genes distinguished cancers, non-cancerous state and precancerous conditions.	2022	(Ref. 124)
**Neurodegenerative diseases**						
Toden *et al*.	Alzheimer's disease	Plasma	RNA-Seq	Demonstrated the feasibility of cf-mRNA-based Alzheimer's disease diagnosis.	2020	(Ref. 125)
Yan *et al*.	Alzheimer's disease	Plasma	RNA-Seq	Identified PHGDH as a potential biomarker for Alzheimer's disease.	2020	(Ref. 126)
Cisterna-Garcia *et al*.	Alzheimer's disease	Plasma	RNA-Seq	Identified cf-mRNA biomarkers with high specificity to Alzheimer's disease compared to other neurogenerative diseases.	2023	(Ref. 127)
**Liver diseases**						
Chalasani *et al*.	Non-alcoholic fatty liver disease	Serum	RNA-Seq	Demonstrated the feasibility of cf-mRNA-based diagnostic biomarker for liver fibrosis.	2021	(Ref. 128)
Chalasani *et al*.	Primary sclerosing colitis	Serum	RNA-Seq	Demonstrated the feasibility of cf-mRNA-based diagnostic biomarker for primary sclerosing colitis.	2023	(Ref. 129)

### Prenatal biomarkers

Non-invasive, prenatal disease diagnosis has been a key research area that has extensively utilized cf-mRNAs. Three years following their initial discovery of cell-free fetal DNA (Ref. 130), Lo *et al.* identified fetal-specific Y-chromosome zinc-finger protein mRNA from maternal plasma in 2000 (Ref. 112). In 2014, Koh *et al.* utilized microarray and RNA-sequencing platforms to profile cf-mRNA transcriptome of women during trimesters of pregnancy and discovered that tissue-specific fetal transcripts can be quantified in maternal blood (Ref. 11). Furthermore, a longitudinal study profiling cf-mRNA from the plasma of 60 pregnant women demonstrated that alterations in the plasma transcriptome can be used to monitor inflammatory responses and microbial infections throughout pregnancy (Ref. 113). These studies provided a blueprint for the clinical utilization of cf-mRNA profiling in prenatal diseases and paved the way for subsequent studies.

Over the last 5 years, several large clinical investigations utilized cf-mRNA profiling to develop and establish non-invasive biomarkers for prenatal diseases. In 2018, a panel of cf-mRNA transcripts was identified that predicted gestational age and distinguished pregnant women who would deliver prematurely (Ref. 114). The concept of this study was further bolstered in a recent investigation where cf-mRNA profiles of plasma samples were compared from pregnant women who did or did not have preterm birth – 25 cf-mRNA transcripts were found to associate with an increased risk of preterminal birth (Ref. 131).

Various research groups have also utilized cf-mRNA profiling to develop predictive biomarkers in preeclampsia. A study which investigated the plasma cf-mRNA profiles of 199 pregnant mothers at 5–16 weeks of the pregnancy, compiled an 18 gene cf-mRNA signature that robustly discriminated between normotensive and preeclamptic mothers (Ref. 116). Similarly, by utilizing plasma samples from 1840 pregnancies, researchers built a cf-mRNA-based diagnostic classifier that was able to robustly predict pre-eclampsia approximately 14-weeks prior to the delivery (Ref. 117). A different study performed cf-mRNA-sequencing on 40 pregnancies at the time of preeclampsia diagnosis with 73 gestational age-matched controls, and identified a 49-gene classifier capable of discriminating early-onset preeclampsia from controls with 85–89% accuracy (Ref. 115). Circulating transcripts that are associated with tissue-specific fetal maturation have also been found with cf-mRNA profiling on the amniotic fluid of rhesus macaque (Ref. 132). Together, these studies demonstrate that molecular dysregulation of the cf-mRNA secretome in mothers can reflect prenatal disorders. While a genome-wide association study has identified genetic variants associated with preterminal birth (Ref. 133), these markers are inadequate to be accurately used for prognosis. Considering that transcriptional alterations are dynamic and reflect acute physiological events in both the mother and fetus(es), profiling of bioactive cf-mRNA appears to be more suitable to predict preterminal birth than cfDNA-based biomarkers.

### Cancer biomarkers

Another research area where cf-mRNA has been used prominently is non-invasive cancer detection. Cancer-specific cf-mRNA alterations were first discovered in 1999 (Refs 7, 8), where cancer-associated viral RNA and tyrosinase mRNA were identified in the circulation of cancer patients. Several years later, mRNA of telomerase components was quantified in the plasma of cancer patients (Refs 134, 135). While telomerase activity is a well-recognized characteristic of cancer cells, a large portion of cancers do not express telomerase, which restricts its robustness as a diagnostic biomarker in oncology (Refs 136, 137). Additionally, in 2002 Lo *et al.* identified significantly higher GAPDH cf-mRNA concentrations in patients with hepatocellular carcinoma compared to that of healthy controls (Ref. 138).

Although an individual gene cf-mRNA biomarker is attractive, the heterogeneity of cancer is now recognized as a major obstacle for the development of accurate cancer biomarkers, and tissue-based transcriptional gene signatures that incorporate large sets of genes have gained favour for subcategorizing cancers (Ref. 139). Importantly, this approach has been adopted to identify cf-mRNA-based biomarker candidates. For example, in prostate cancer, candidate transcripts were pre-selected using the Cancer Genome Atlas tissue-profiling database, and a qPCR-based, three-gene high-risk patient stratification panel was developed (Ref. 122). Similarly, gene-expression comparison between melanoma and healthy tissues resulted in the identification of six markedly elevated genes in melanoma, with four out of six genes (*KPNA2*, *DTL*, *BACE2* and *DTYMK*) being validated in the plasma of both melanoma subject and healthy controls using targeted digital droplet PCR (Ref. 140).

Separately, several groups have utilized cf-mRNA sequencing to identify target genes that are dysregulated in cell-free transcriptome of cancer subjects. A recent study conducted cf-mRNA profiling on stage III breast and lung cancers, identifying several cf-mRNA genes that are tissue and cancer specific (Ref. 123). Plasma cf-RNA-Seq profiling was also conducted on eight hepatocellular carcinoma and ten multiple myeloma subjects identifying several tissue-specific, cancer biomarker candidates (Ref. 124). A subset of these tissue specific, oncogenic, cf-mRNA genes was validated using qRT-PCR. Additionally, a cf-mRNA-sequencing protocol was used to monitor transcriptional changes in haematological cancer subjects during bone marrow ablation and repopulation (Ref. 121). Investigators were able to demonstrate that cf-mRNA is multifaceted and able to reflect cancer recurrence as well as acute, disease-associated physiological changes. Intriguingly, another paper demonstrated that the cf-mRNA profile of subjects with five different cancer types all deviated from healthy controls and that the microbe-derived cf-mRNA expression also differed between the groups (Ref. 141). With cancer-associated perturbations measured in the microbiome, this study provides a newly recognized source of oncologic cf-mRNA signal. In addition, a mouse xenograft model demonstrated that tumour released cf-mRNA from human cancer cell lines is distinguishable from murine host RNA, and that the RNA expression from specific tumour tissue and cell lines correlates with cf-mRNA signals (Ref. 142). This confirms that tumour-derived mRNA is quantifiable in the circulation.

Considering higher survival rates for early-stage cancers, precise prognosis and/or diagnosis of early-stage cancers through blood-based diagnostic biomarkers is highly appealing to improve patient outcomes. Most advanced cancers can be accurately diagnosed using cfDNA biomarkers (Ref. 143), however, early-stage cancers generate lower levels of detectable tumour mutations limiting cfDNA-based biomarker sensitivity. Considering that transcriptional dysregulations are more prevalent than genomic alterations, cf-mRNA appears to be a well-suited vehicle for early-stage, non-invasive cancer biomarkers (Ref. 144). As cf-mRNA gains momentum, a key confounding variable to address may be the extent to which cf-mRNA is released from the dysplastic or malignant tissue.

### Other disease biomarkers

Along with prenatal care and oncology, difficulties associated with conducting brain biopsy augment the necessity for non-invasive molecular biomarkers in many neurodegenerative pathologies. The most recent transcriptome-wide comparison of major tissues, organs and blood cells revealed that the brain has the second most tissue-enriched transcripts following the testis (Ref. 145). Interestingly, brain-specific, Alzheimer's disease (AD) associated, and glioblastoma-specific cf-mRNA transcripts have been identified in the circulation (Ref. 11). A subsequent team of researchers sequenced the transcriptome of 200 plasma samples derived from AD and aged matched subjects (Ref. 125), discovering that the dysregulation of cf-mRNA genes in the plasma of AD patients resembles AD brain tissue transcriptional alterations. This team was also able to develop a promising AD diagnostic classifier (Ref. 125). Additionally, a 15-year longitudinal study of subjects aged 70 years or older utilized RNA-sequencing on the plasma of AD subjects and identified PHGDH cf-mRNA as a potential pre-symptomatic indicator (Ref. 126). Currently, the assessments of molecular alterations in neurological disorders can only be conducted on the post-mortem brain tissues and animal models, so non-invasive means to assess molecular dysregulation in the brain will be required to advance patient care, patient management, and precision medicine approaches. With emerging evidences that EVPs cross the blood–brain barrier carrying genomic material from brain into circulation (Ref. 146), cf-mRNA is growing in promise as a non-invasive biomarker analyte.

The liver's key role in the regulation of blood flow and its inherent size coincides with the extracellular transcriptome's high proportion of liver-derived genes (Ref. 121). This high abundance in the circulation makes liver diseases attractive, early targets for cf-mRNA biomarker development. Using an animal model of inflammation, gene-expression profiles have been compared between plasma cf-mRNA and major internal organs, highlighting that chemically induced inflammatory responses in the liver highly correspond with plasma cf-mRNA profile (Ref. 147). This study supports future efforts to both understand and transit from animal models to human disease. Another study performed cf-mRNA sequencing on 300 serum samples from patients with non-alcoholic fatty liver disease and healthy subjects to develop a diagnostic classifier for advanced liver fibrosis (Ref. 128). The same cf-mRNA sequencing platform and classifier was then subsequently used for 50 primary sclerosing cholangitis subjects (Ref. 129). The highly abundant liver-specific transcripts were able to provide discrimination of primary sclerosing cholangitis subjects from healthy controls as well as other liver diseases. This monitoring of transcriptional activity from the liver study was then independently supported using a modified small RNA sequencing protocol to assess circulating cf-mRNAs and lncRNAs of patients with liver injuries (Ref. 148).

## Concluding remarks and future directions

With continued advancement in quantification methods for the cf-mRNA transcriptome and improved characterization of EVPs, cf-mRNA will likely play a major role in the development of non-invasive, clinically relevant biomarkers for health and disease management. One of the key goals to develop robust cf-mRNA platforms is to understand the molecular composition and origin of EVPs, as researchers will no doubt delve into characterizing specific carrier types and subsequently utilize their profiles to develop disease-specific biomarkers. The plethora of extracellular RNA carriers and their innate heterogeneity including cell-type disparity as well as overlapping size, density, and constituents has precluded discrete purification and characterization. Many previously presumed proteins ascribed to specific EVs are instead associated with other small EVs or non-vesicular components, including EPs, which indicates there is either a coincidence of enriched cargo in various EVPs or that conventional isolation methodologies have been unable to differentiate distinctive EVPs (Ref. 37).

Immense work has been performed over the last decade to improve methods of isolation and purification of EVPs (Refs 33, 36, 40). Currently, key purification methods include exosomal pelleting, iodixanol gradient, serial ultracentrifugation, direct immunoaffinity capture and asymmetric flow field-flow fractionation. These advancements in isolation techniques, although helpful for progress in the field, will require careful standardization in the future. Several experimental factors that impact purification processes include, rotor types, centrifugation times, and EVP-specific protein markers (Ref. 149). Furthermore, disparate *in vitro* experimental conditions that alter EVP release rates and cargo (Ref. 21), varying mRNA concentrations between different cell lines (Refs 60, 72), and unresolved nomenclature all contribute to confounding conclusions. Additionally, mRNA is often neglected or undetected due to its paucity in the extracellular compartment. As EVPs are now realized to be central informants in the cf-mRNA secretome, it is crucial to completely describe the panoply of extracellular nucleic acid carriers to develop clinically relevant biomarkers and therapeutics.

**Figure 4.**
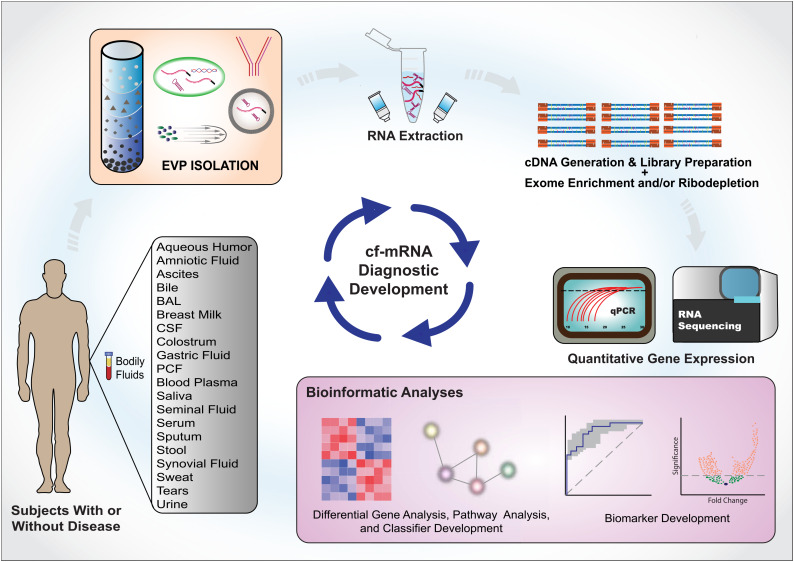
Cf-mRNA-based liquid biopsy biomarker development pipeline. Body fluid collection (bottom left corner). Types of fluids that can extract cf-mRNA are listed in the box. Selection of specific extracellular vesicles and particles (EVPs) via EVP isolation/purification (top left corner). RNA extracted from directly from body fluids or specific EVPs (top middle). cDNA conversion and sequencing library preparation (Top right). Isolated RNA samples are converted to cDNA and subsequently sequencing libraries are generated. These libraries can undergo exome hybridization process to enrich for messenger RNA or depletion of unwanted transcripts (such as ribosomes). Data generation and analysis (bottom right). Bioinformatic analyses are performed on data generated by next-generation sequencing, qPCR or other quantification methods. Analyses such as differential expression analysis and pathway analysis are conducted to identify key genes and pathways that are dysregulated in the targeted disease. Finally, machine learning approaches will be used to combine the key molecular features and develop robust diagnostic classifiers.

As a granular view of EVP mechanisms is needed for identifying therapeutic and diagnostic targets, understanding EVP biogenesis and loading will bring added value to future interventions (Ref. 37). For instance, knowledge of specific cell internalization mechanisms will be essential in order to capture particular EVP subsets from bulk fluids and improve disease-specific signals during analysis – a key challenge to-date (Refs 2, 28). In parallel with and in advance of delineating the biogenesis and cargos of each RNA carrier, surveys of the entire cf-mRNA compartment with deconvolution of signals using tissue and cell type-specific references can advance deciphering this bioactive compartment. Additionally, because of this high dimensionality and connectivity of the exRNA carriers, studies integrating multiple fields and methodologies to characterize EVPs will translate discovery research to clinical applications (Ref. 2). Progress in utilizing large sequence datasets and the development of novel machine learning approaches, for example, promise to advance development of generalizable non-invasive molecular biomarkers (Fig. 4).

In conclusion, cf-mRNA profiling along with improved characterization of EVPs and ribonucleoprotein complexes will contribute to advancements in the diagnostic field. Concomitantly, tailored approaches considering the biological questions and types of extracellular cargo continue to be developed such as transcriptome deconvolution techniques that reveal low-dimensional latent substructures of high-dimensional extracellular cargo data (Ref. 150). Transcriptome surveillance will likely gain more attention in the coming years as cf-mRNA provides significant advantages including cell-type and pathway-specific gene-expression quantification, informative data on impacted biological pathways, and temporal measurements that are unobtainable using other ribotypes. Decoding bioactive signals of the RNA secretome combined with critical translational science and medicine practices promise to lead to diagnostic and therapeutic advancements that will change how populations are screened, patients are managed and treatments are selected and monitored.
